# Unraveling the Ethical Enigma: Artificial Intelligence in Healthcare

**DOI:** 10.7759/cureus.43262

**Published:** 2023-08-10

**Authors:** Madhan Jeyaraman, Sangeetha Balaji, Naveen Jeyaraman, Sankalp Yadav

**Affiliations:** 1 Orthopedics, ACS Medical College and Hospital, Dr. MGR Educational and Research Institute, Chennai, IND; 2 Orthopedics, Government Medical College, Omandurar Government Estate, Chennai, IND; 3 Medicine, Shri Madan Lal Khurana Chest Clinic, New Delhi, IND

**Keywords:** secure multiparty computation, homo-morphic encryption, healthcare, large language models, chatgpt, artificial intelligence (ai)

## Abstract

The integration of artificial intelligence (AI) into healthcare promises groundbreaking advancements in patient care, revolutionizing clinical diagnosis, predictive medicine, and decision-making. This transformative technology uses machine learning, natural language processing, and large language models (LLMs) to process and reason like human intelligence. OpenAI's ChatGPT, a sophisticated LLM, holds immense potential in medical practice, research, and education. However, as AI in healthcare gains momentum, it brings forth profound ethical challenges that demand careful consideration. This comprehensive review explores key ethical concerns in the domain, including privacy, transparency, trust, responsibility, bias, and data quality. Protecting patient privacy in data-driven healthcare is crucial, with potential implications for psychological well-being and data sharing. Strategies like homomorphic encryption (HE) and secure multiparty computation (SMPC) are vital to preserving confidentiality. Transparency and trustworthiness of AI systems are essential, particularly in high-risk decision-making scenarios. Explainable AI (XAI) emerges as a critical aspect, ensuring a clear understanding of AI-generated predictions. Cybersecurity becomes a pressing concern as AI's complexity creates vulnerabilities for potential breaches. Determining responsibility in AI-driven outcomes raises important questions, with debates on AI's moral agency and human accountability. Shifting from data ownership to data stewardship enables responsible data management in compliance with regulations. Addressing bias in healthcare data is crucial to avoid AI-driven inequities. Biases present in data collection and algorithm development can perpetuate healthcare disparities. A public-health approach is advocated to address inequalities and promote diversity in AI research and the workforce. Maintaining data quality is imperative in AI applications, with convolutional neural networks showing promise in multi-input/mixed data models, offering a comprehensive patient perspective. In this ever-evolving landscape, it is imperative to adopt a multidimensional approach involving policymakers, developers, healthcare practitioners, and patients to mitigate ethical concerns. By understanding and addressing these challenges, we can harness the full potential of AI in healthcare while ensuring ethical and equitable outcomes.

## Introduction and background

In the ever-evolving landscape of healthcare, the advent of artificial intelligence (AI) has emerged as a transformative force, promising unparalleled advancements in patient care. The use of AI has enhanced clinical diagnosis, predictive medicine, patients' data and diagnostics, and clinical decision-making [1]. The tools utilized to provide data processing and reasoning capabilities at par with those of human intelligence in machines and software include machine learning (ML), large and often unstructured datasets, advanced sensors, natural language processing (NLP), and recently, large language models (LLMs). ML, a subset of AI, enables algorithms to learn patterns from data without explicit programming. Deep learning is a subset of ML that uses algorithms known as artificial neural networks to learn from data and make predictions or decisions. It is particularly useful for tasks such as image and speech recognition [2]. The study of the application of natural language in interactions between computers and people is known as NLP, a branch of computer science and AI. To analyze, comprehend, and produce human language, computer methods and algorithms are used [3,4]. Developed by OpenAI, ChatGPT is a sophisticated LLM based on the GPT-3.5 architecture, extensively trained on vast text data to generate human-like responses to user inputs. Its applications in medical practice, research, and education are promising. Notable competitors in this field include Microsoft Bing and Google's Bard [5-7].

The opportunity to integrate AI into healthcare has been taken by medical specialties where images are essential, such as radiology, pathology, or oncology, and significant research and development efforts have been made to translate the potential of AI to therapeutic applications [8-10]. Additionally, ML is being used to analyze neuroimaging data to help with the early identification, prognosis, and treatment of disorders that pose a threat to brain health [11]. Segar et al. developed ML models that integrated social determinants of health, outperforming traditional logistic regression models [12]. AI applications in the field of mental healthcare have the potential to offer advantages, including new treatment modalities, chances to connect with underserved communities, and improved patient response [13]. Ashburner et al. analyzed narrative data from electronic health records using NLP to enhance the prediction of incident atrial fibrillation (AF) risk [3]. NLP can also be used to supplement symptom assessment in clinical and research settings for schizophrenia. It uses information that is likely to be more related to impaired brain processes such as impaired connectivity, information processing, and reward processing [4]. ChatGPT's capabilities make it a valuable tool for medical education through curriculum development, simulated training, and language translation. Additionally, it can assist in information retrieval for research purposes and potentially improve the precision and speed of medical recording in clinical settings [14,15].

However, as we embrace this revolutionary technology with open arms, we must also confront the profound ethical concerns that accompany its integration into our healthcare systems. AI advancement has the potential to make healthcare inequality more challenging. Given the many benefits of AI and the need to mitigate any potential negative effects, it is imperative to understand and handle the ethical issues in depth. To mitigate the legal and ethical issues related to AI in healthcare, a multidimensional approach encompassing policymakers, developers, healthcare practitioners, and patients is essential [16].

## Review

Ethical challenges and AI

Research on the ethical concerns related to the use of AI in healthcare has identified key ethical issues and related subtopics that require consideration. These issues, along with the difficulties posed by the recent development of publicly accessible LLMs, are discussed in this review. The primary concerns include privacy, transparency, trust, responsibility, bias, cybersecurity, and data quality [14,16-18]. 

Privacy

In the realm of data-driven healthcare, privacy emerges as a critical challenge, given the utilization of ML and deep learning systems to make predictions using users' data. Patients trust healthcare professionals to protect their private information, including sensitive information like age, sex, and health data [19-23]. Big data use in healthcare creates many privacy issues. Among these is the moral conundrum posed by the unauthorized use of personal data in predictive analytics. The loss of control over data access is a key concern since it could have a serious psychological impact on patients if their private health information is exposed. Additionally, the availability of private databases involving genetic sequences and medical history could hinder the collection of data and the advancements in medical tests. Furthermore, some businesses may justify withholding data under the guise of preserving privacy, making data sharing challenging [20].

In the context of wearable devices and the Internet of Medical Things (IoMT) in healthcare, a plethora of security and privacy vulnerabilities exist, which pose a significant risk to sensitive data, including passwords. Denial-of-service and ransomware attacks have the potential to result in life-threatening circumstances. Users consequently voice concerns about the technology's vulnerabilities and the use of their data [22]. Health information management (HIM) practices such as automated medical coding, information capture, data management, and governance are significantly impacted by AI technologies. Moreover, AI-based applications have a direct impact on patients' confidentiality and privacy [21]. Finding the ideal balance between data access restrictions and the possible problems they can reduce is ultimately a crucial challenge.

Priyanshu et al. examined how ChatGPT can adhere to privacy-related policies and privatization mechanisms by providing outputs that comply with regulations like the Health Insurance Portability and Accountability Act (HIPAA) and General Data Protection Regulation (GDPR). The study involves instructing ChatGPT to generate outputs that are compliant and observing the extent of personally identifiable information (PII) omission from the responses. The objective is to explore ways to limit input copying and regurgitation in ChatGPT's responses by incorporating add-on sentences into prompts. This approach aims to induce privacy through sanitization of ChatGPT's generated responses while adhering to relevant data protection regulations. ChatGPT retains PII verbatim in 57.4% of cover letter summaries, with varying rates across subgroups. When instructed, it omits PII significantly, showing potential for privacy compliance [24].

To preserve privacy in healthcare applications, proposed solutions include homomorphic encryption (HE), allowing computations on encrypted data while preserving data privacy and enabling secure processing without the need for decryption. Secure Enclave (SGX), a hardware-based security technology, safeguards the confidentiality and integrity of code and data during execution within a secure enclave, protecting sensitive computations from potential threats. Secure multiparty computation (SMPC) distributes computations across multiple parties, preventing individual access to others' data. SMPC can be achieved through garbled circuits and secret sharing, enabling secure collaborative computation on sensitive data. These solutions are crucial in healthcare, where strict regulations protect patient privacy and data. By implementing these methods, sensitive information can be processed efficiently and securely while ensuring compliance with data protection standards in healthcare settings [22,23].

Transparency and trust

Transparency has emerged as a significant ethical concern, especially in complex *black box* AI systems that are highly efficient but lack clarity in their decision-making processes. Striking a balance between accuracy and explainability is crucial, especially in high-risk decision-making situations [25-28]. Despite existing guidance for transparent reporting, poorly reported medical AI models are still common, and the transparency required for trustworthy AI remains unfulfilled. Using a survey to prompt structured reporting on intended use, AI model development and validation, ethical considerations, and deployment constraints based on current guidelines, a paper presents a framework to measure transparency and trustworthiness in medical AI tools. The framework was piloted with three medical AI tools, and the assessment revealed reporting gaps and lowered the degree of trustworthiness, indicating compliance gaps with ethical guidelines [29].

Explainability is the ability of an AI-driven system to provide a person with an understanding of why it arrived at a certain prediction or decision. From a medical standpoint, it is essential to differentiate between two levels of explainability in AI systems. The first level pertains to understanding how the system reaches its conclusions in a general sense, while the second level involves explaining the training process that enables the system to learn from examples and produce outputs. From a technological viewpoint, achieving explainability is crucial, considering both its implementation and the advantages it offers during development. Legally, informed consent, certification, approval, and liability are critical aspects related to the explainability of medical devices [30]. In a survey focusing on explainable AI (XAI) applications in healthcare and medical imaging, various XAI types are summarized and categorized. The XAI types can be classified as model-specific techniques, which are tailored for a specific model type and cannot be applied to other models, and model-agnostic techniques, which have no specific requirements and can be used with a wide range of XAI models. The paper also highlights specific algorithms used to enhance interpretability in medical imaging. These algorithms, such as Local Interpretable Model-Agnostic Explanations (LIME), Backpropagation, Class Activation Mapping, and Layer-wise Relevance Propagation, fall under post hoc interpretation techniques. These techniques provide interpretable information about the model through external methods after its analysis [31]. Failure to prioritize explainability in clinical decision support systems can jeopardize core ethical values in medicine and may have adverse effects on both individual and public health [25,30,31].

Cybersecurity

Cybersecurity is the practice of preventing unauthorized access, theft, damage, or other harmful attacks on computer systems, networks, and digital information. Security breaches can be concealed by AI systems' incapacity to be explained and interpreted [27]. Open-source intelligence (OSINT) utilizes publicly available data from various sources, raising implications in areas like national security, political campaigns, the cyber industry, criminal profiling, societal issues, cyber threats, and cybercrimes [32]. The COVID-19 pandemic has introduced novel cybersecurity challenges, leading to the emergence of new themes in this field. Healthcare and cyber resilience are among the newly added cybersecurity themes that have been extensively researched in peer-reviewed literature. On the other hand, non-peer literature highlights the observation of newer forms of cyberattacks, such as social engineering and side-channel attacks [33]. These developments reflect the evolving nature of cybersecurity concerns. Stanfill and Marc highlighted the need for data security at various points, such as when collecting data from devices, transferring data between devices, storing data, and using the data [21].

In a systematic review, the utilization of semi-supervised learning (SSL) with cybersecurity data repositories is explored to construct robust models for computer security and cybersecurity systems. SSL is a specific type of ML that requires only a limited number of labels or may work with partially labeled data to build these models. It is crucial that the datasets used in developing these models for cybersecurity accurately represent real-world data to ensure their effectiveness and relevance [34]. As a fundamental framework for understanding the links and interdependencies among various cybersecurity components from a complete cybersecurity perspective, Grobler et al. offered the 3U's of cybersecurity: user, usage, and usability. The study demonstrates a paradigm shift away from a functional and usage-centric approach to cybersecurity and toward a user-centered strategy that emphasizes the human aspects of users. This user-centered cybersecurity strategy takes into account the human factor, which has the potential to boost the effectiveness of cybersecurity measures [35]. However, since cybersecurity is a constantly evolving challenge, it is essential to develop new strategies to prevent it.

Responsibility

AI responsibility attribution poses significant questions regarding who or what should be held liable for the outcomes of AI actions [36-38]. Some papers explore human responsibility concerning AI systems, emphasizing meaningful control and due diligence and caution against fully automated systems in medicine. They assert that *moral agency* is a human attribute absent in AI due to the lack of autonomy and sentience [38]. Others advocate for examining the causal chain of human agency, including interactions with technical components like sensors and software, to determine accountability. The temporal dimension is vital in considering AI's development, application, and maintenance, along with its interactions with other technical elements. Furthermore, since people might not fully understand how AI is involved or what their function as users is, the extent of end users' voluntary and informed use of AI is called into doubt [36]. Responsibility diffusion occurs when there are multiple options and several agents involved, making it challenging to attribute responsibility clearly. The concept is exemplified through the case of an AI-driven *digital tumor board*, where clinical decision-making is altered, leading to a diffusion of responsibility among various parties [37]. Addressing the structural and temporal connections between humans and technological elements, ensuring the transparency and traceability of AI systems, and comprehending how technology interfaces contribute to AI-related issues are all necessary for addressing these responsibility attribution challenges [36]. In the context of ChatGPT, the question of whether AI tools should be credited as authors sparks a debate among experts. Some argue that AI tools lack the capacity to make decisions or contribute to research in the same way human authors do, making authorship inappropriate. On the other hand, proponents contend that AI tools can play a significant role in generating ideas and assisting in the writing process, warranting appropriate acknowledgment and credit in the authorship of papers [39]. The issue revolves around the level of contribution and autonomy that AI tools bring to the research process and the ethical considerations surrounding their recognition in academic and scientific work.

Shifting from data ownership to data stewardship is crucial to ensure responsible data management, safeguard patients' privacy, and adhere to regulatory standards. Data stewardship involves governance and protection of data, including determining access and sharing permissions, ensuring regulatory compliance, and facilitating collaborations and data exchange for research and technological advancements. Tech giants combine AI and healthcare for growth, using personal health data for personalized features. To enhance privacy and data security in federated learning, a decentralized approach is being adopted to train ML models. Data stewards play a vital role in making data available, accessible, and retrievable while upholding privacy and compliance standards [18].

Bias

AI algorithms can be influenced by biases present in healthcare data. Biases in healthcare data can have an impact on AI algorithms. In addition to well-known study biases like blinding and sampling, we also need to identify implicit and explicit biases in the healthcare system. Large-scale data used to train AI systems may be impacted by these biases. Clinical decision-making can be influenced by factors like clinical trial eligibility requirements and implicit biases present in real-world treatment decisions, which can affect the predictions given by AI [26]. AI can lead to healthcare inequities through biased data collection, algorithm development, a lack of diversity in training data, transparency, and research teams, requiring efforts to address biases and promote equitable outcomes. Particularly with regard to demographic traits like sex and ethnicity, there is growing recognition of the detrimental effects of model bias. Studies have also revealed poorer implementation rates for specific diseases in rural areas, racial and ethnic minority groups, those without insurance or with inadequate insurance, as well as individuals with lower education and income [9,40-42].

Khoury et al. advocated a public-health approach to address inequalities. Targeted interventions, policy development, and ensuring ethical and the establishment of efficient delivery systems should be the main goals of public health initiatives. It is essential to involve communities, build coalitions, improve genetic health literacy, and promote diversity in the workforce [40]. The potential of AI to address health disparities in both high- and low-income countries (HICs) and low- and middle-income countries (LMICs) is highlighted in a scoping review; however, it is acknowledged that adoption of AI requires adequate infrastructure. AI has proven to be helpful in finding racial disparities in cancer outcomes and examining the influence of race and socioeconomic position on health outcomes in oncology [43].

Data quality

Convolutional neural networks (CNN) have gained popularity in image-related tasks, and their application has extended to non-imaging data through modern ML techniques. CNNs can be used for a variety of activities outside of conventional imaging tasks by transforming non-imaging input into images. This gives healthcare practitioners the opportunity to train hybrid deep learning models using multi-input/mixed data models that incorporate different patient information, such as genetics, imaging, and clinical data. In comparison to relying just on one data type, the integration of various data types offers a comprehensive and multiperspective picture of patient data. This strategy shows potential for improving the caliber of data generated by AI [44].

The term *hallucinations* refers to a noteworthy characteristic of LLMs that arises when the model produces inaccurate or misleading information that appears to be factual or coherent. As a result, language models could produce answers or data that is entirely fictitious or unsubstantiated by actual data. For language models to be reliable and trustworthy, it is essential to recognize and deal with these hallucinatory tendencies, particularly in the realm of healthcare, where precise and true information is critical [45]. A paper points out that as automation advances, the ability of HIM professionals to identify data patterns becomes essential, requiring additional skills in data analysis tools and techniques. With their in-depth knowledge of healthcare data sources and origins, HIM professionals are well-positioned to adapt to evolving AI technologies and take on new roles [21].

## Conclusions

The integration of AI in healthcare presents multifaceted ethical challenges that demand meticulous consideration. This review highlights complex issues and potential risks associated with AI implementation in healthcare. Privacy emerges as a critical concern, with data-driven healthcare relying on ML and deep learning systems that utilize users' data for predictions, necessitating a delicate balance between data protection and access. Transparency and trust are crucial for successful AI adoption, particularly in high-stake decision-making contexts where the lack of clarity in AI's decision-making processes can breed skepticism and distrust. Developing frameworks and guidelines for transparent reporting and structured AI model assessment can enhance the trustworthiness and the ethical use of AI in medical applications. Cybersecurity concerns are of utmost importance to safeguard patients' safety and data integrity, urging the implementation of security measures like HE and Secure Enclave to protect against cyber threats. Responsibility attribution in AI remains complex and evolving, requiring a balance between human agency and AI capabilities, while shifting from data ownership to data stewardship can ensure responsible data management and privacy protection. Addressing biases in AI algorithms and data collection is essential to promoting equitable healthcare outcomes, as biases can impact AI-generated predictions and exacerbate healthcare inequities. Leveraging CNNs and multi-input/mixed data models can improve data quality and provide a comprehensive view of patients' information, enhancing the accuracy of AI-generated insights. The phenomenon of *hallucinations* in large language models necessitates rigorous validation and fact-checking to ensure the accuracy and reliability of AI-generated outputs. Collaboration among researchers, healthcare professionals, policymakers, and technology experts is essential to overcome ethical challenges and promote responsible AI use in healthcare. By developing comprehensive guidelines, regulatory frameworks, and technical solutions prioritizing privacy, transparency, and fairness, AI can revolutionize healthcare delivery and improve patient outcomes while upholding ethical principles.
